# Psychological Empowerment of Nurses Working in Pediatric Units in Saudi Arabia

**DOI:** 10.3390/healthcare10122374

**Published:** 2022-11-26

**Authors:** Manal F. Alharbi, Reham Z. Alrwaitey

**Affiliations:** 1 Maternal & Child Health Nursing Department, College of Nursing, King Saud University, Riyadh P.O. Box 642, Saudi Arabia; 2 Nursing Education Administration, Department, Maternity & Children Hospital, King Salman Bin Abddulaziz Medical City, Ministry of Health, Madinah 36422, Saudi Arabia

**Keywords:** psychological empowerment, pediatric units, nurses, Saudi Arabia

## Abstract

**Background**: This study investigated the psychological empowerment (PE) of nurses in pediatric units and examined the significant difference between the level of PE and the personal work profiles of nurses working in pediatric units in Saudi Arabia. **Methods**: A quantitative descriptive study design was employed between May and July 2020 using a self-administered electronic survey that collected personal work profile data and applied the Psychological Empowerment Scale. The data were analyzed using SPSS 22.0 software for descriptive statistics, *t*-tests, and ANOVA. **Results**: Most of the study participants (69.6%) were non-Saudi nurses, and 71.9% worked in specialized areas. The mean PE score of the four dimensions across the 12 items was high. The mean score for the dimension meaning was the highest, whereas the lowest mean score was for the dimension self-determination. Nurses from different nationalities had significantly different PE levels. **Conclusions**: Expatriate nurses were more psychologically empowered. The findings will be of interest to all who wish to attract and retain local nurses by fostering PE in the work environment and involving nurses in decision-making processes, thus enabling them to act as leaders for increased work motivation.

## 1. Introduction

Among the most significant groups of professional nurses, pediatric nurses play a vital role in the determination and provision of quality healthcare to children. As nurses face a number of challenges, empowerment is a major factor that affects their performance [1]. Psychological empowerment (PE) can be traced back to and seen from the perspective of motivational theory; the theory of PE was first developed by Conger and Kanungo [2] to provide an analytical meaning of the PE construct by integrating diverse approaches in both management and psychology literature. The subsequent work of Thomas and Velthouse [3] identified four cognitive elements of empowerment, namely, meaning, competence, self-determination (choice), and impact [4]. Later on, Spreitzer [5] developed a measure from the four components that Thomas and Velthouse had identified and defined PE as a motivational construct. The four dimensions of empowerment may help individuals feel more in control [6]. The overall impact of experienced empowerment will be decreased if a single dimension is lacking but not completely reduced. Additionally, PE is defined as an elevated concept of an essential motivational task that manifests in four sets of elements that reflect one’s orientation to one’s work role [6]. According to the study by Wang and Liu [6], the meaning dimension reflects the congruence between a job’s requirements and an individual’s beliefs. Competence indicates the individual’s confidence in their capability to accomplish work tasks. Self-determination refers to one’s autonomy to initiate and regulate work processes. Lastly, impact refers to the degree to which one can influence the activities and outcomes of the organization [6].

PE in the healthcare field is directly associated with the quality of nursing care [7]. Evidence suggests that PE is one of the most important factors for autonomy, self-confidence, and independence, thereby improving organizational and patient outcomes [5]. As a result, nurses can provide quality care. Furthermore, PE has a positive influence on individuals as well as organizational outcomes [8]. PE has also been shown to increase the effectiveness of staff members and raise their performance levels within a Western context [8]. A recent systematic review and meta-analysis exploring the relationship between psychological empowerment (PE) and job satisfaction among nurses reported that the PE of nurses affected job satisfaction and, in turn, reduced their turnover [7]. A systematic review analyzing studies that examined the relationship between nurses’ empowerment and job satisfaction in their work environment revealed a significant positive correlation between empowerment and job satisfaction among nurses [9]. Numerous studies have attempted to explain the mediating effect of PE. For instance, in China, positive relationships among PE, job satisfaction, and organizational commitment were reported [10]. A cross-sectional study reported that PE had mediating effects on burnout [11]. Additionally, the study by Fan et al. [12] reported that PE appears to be positively correlated with job engagement and has a mediating effect on the relationship between perceived work environment and work engagement among Chinese nurses. In New Zealand, a mixed-method approach applied to data obtained from emergency nurses revealed that PE may improve their ability to act as clinical leaders [13]. In Egypt, a correlational study reported statistically significant relationships between PE and perceived autonomy among nurses [14]. A study with a quasi-experimental design using a PE program for oncology nurses reported increased scores for PE and decreased scores for burnout in Turkey [15]. A recent study from Iran reported moderately high levels of PE, and the highest mean value was observed for the subscale competence, with a significant correlation between PE and moral courage [16]. Another recent study reported the mediational roles of PE and work-life quality on the burnout experiences of nursing staff in Malaysia [17]. Another study reported that PE had a significant and positive influence on nurses by enhancing their compliance and participation in safety measures [18]. The available literature that provides considerable evidence so far is, however, based on data from international contexts. Empowerment has not been investigated in a Saudi Arabian context [19].

In Saudi Arabia, the national nursing workforce represents 36.5% of the country’s healthcare staff, with continuous growth being shown in the number of Saudi nurses [20]. However, the current number of national nurses is still not sufficient to meet the need for more nursing staff [21]. According to Wang and Liu [6], an insufficient number of nurses may lead to excessive workloads and, in turn, negative outcomes and turnover [22]. In light of the current literature review, nurses’ empowerment has been studied internationally, yet little evidence exists in the context of nursing in Saudi Arabia. Current evidence that focuses on nurses educated and working in Saudi Arabia and those working in Saudi Arabia who were educated elsewhere needs to be added to the literature. Furthermore, the link between PE and nurses working in pediatric units in Saudi Arabia has not been studied, especially during the COVID-19 pandemic. Hence, the present study aimed to examine the PE status of registered nurses in pediatric units and investigate the relationships between the four dimensions of PE (meaning, impact, self-determination, and competence, as identified by Spreitzer) and the personal work profiles in pediatric units. In the current study, PE dimensions were identified as nurses’ compatibility between job demands and personal beliefs (competence construct), their confidence to accomplish work tasks (meaning construct), their ability to initiate and regulate the work task (self-determination construct), and their care, reflecting on patient and organization outcomes (impact construct) [5,6]. Based on the literature review, the study hypothesized that there are significant mean differences between personal work profiles and psychological empowerment (meaning, competence, self-determination, and impact).

## 2. Materials and Methods

### 2.1. Design and Participants

This study adopted a quantitative descriptive design to efficiently gather a large amount of data at one point in time. Descriptive research is a powerful tool that permits a researcher to accurately describe a research problem [23]. All pediatric nurses from two governmental referral hospitals in the Western Province of Saudi Arabia were included. However, nurses working in managerial offices, non-pediatric nurses, nurses in their probation period, and students on internships were excluded. The current study was based on a non-probability sample. The sample size was calculated using the Raosoft online calculator with a 95% confidence level, 5% margin of error, and 50% response distribution [24]. With an estimated population of 500 pediatric nurses, the required sample size was determined as 218 participants. Considering that the dropout rate was 21%, we recruited 263 pediatric nurses.

### 2.2. Study Instrument

The study was conducted from May to July 2020. Data were collected from the target participants after initial contact with the administrators to describe the research background, explain ethical considerations, and gain access to a list of the participants’ email addresses. Participants were then recruited to answer a self-administered electronic survey that consisted of two parts. Part I assessed the personal work profile (such as age, gender, marital status, nationality, and educational level) and the current working unit (such as specialty area in critical care, emergency, general areas in pediatric medical-surgical wards, current working hours, and years of experience). Part II contained the PE scale (PES) developed by Spreitzer [5]. The PES included 12 items covering the 4 dimensions (meaning, competence, self-determination, and impact) with 3 questions for each dimension. Responses were rated for each dimension on a seven-point Likert scale with anchors from (1) as very strongly disagree to (7) as very strongly agree. The overall scores for each dimension were calculated. A higher score means a higher psychological empowerment perception and is considered a positive characteristic of being more psychologically empowered. The total score was divided by the number of items to calculate the average score for each subscale and the average of all subscales for the overall scale level. A score of 34 or less was considered low empowerment; a score which ranges from 35 to 68 was considered moderate empowerment; and a score of more than 68 was considered high empowerment [14].

### 2.3. Psychometric Properties of the PES

Internationally, this instrument has been used successfully in more than 50 studies. The PES has been adopted in different contexts, including studies conducted in Brazil and Turkey [25,26], and showed suitable adequate validity and reliability when applied in different contexts as well as its original one in the United States [5,25,26]. For the current study, validity was evaluated by using a panel of experts (two faculty members and four clinical nurses) in the practice to check if the study topic was adequately represented in the questionnaire. Cronbach’s alpha coefficient of the 12-item PES described by Spreitzer [5] was 0.843 [25]. In addition, the internal consistency for the PES was established using Cronbach’s alpha coefficient, computed for each subscale. Cronbach’s alpha was considered reliable for the 12 items of the entire scale (α = 0.98) and excellent for the competence (α = 0.94), meaning (α = 0.96), impact (α = 0.93), and self-determination (α = 0.93) subscales. The items showed positive and strong correlations, with the total score ranging between 0.71 and 0.92 (values over 0.71 indicate acceptable items) [27]. Additionally, for inter-rater reliability, the intraclass correlation coefficient (ICC) was (0.81). According to Cicchetti [28], the ICC interrater agreement measures between 0.75 and 1.00 and indicates excellent scores.

### 2.4. Data Analysis

After data collection, the data were checked for quality and completeness. No values were missing. Each survey was coded and scored based on the appropriate scoring method. Data were analyzed using the Statistical Package for the Social Sciences (SPSS), Version (22) from IBM Corp, New York, USA. Descriptive statistics were applied to describe the frequencies, means, and standard deviations of sample variables. The *p*-values < 0.05 were considered significant. Parametric tests such as the independent sample *t*-test and analysis of variance (ANOVA) were employed to examine the relationships among the study variables. The assumptions of parametric tests were assessed to prevent any misleading results in the analysis. Hence, the assumptions of normality and homogeneity of variances were assessed. The assumption of univariate normality was assessed for the PES, and based on the results, the multivariate normality of continuous variables was met. Variables with absolute data values of skew index <3.0 and kurtosis index <10.0 were considered normally distributed [29]. Furthermore, the assumption of homogeneity of variances was met using Levene’s test of equality of variances.

## 3. Results

### 3.1. Characteristics of the Study Population

The demographic data revealed that 263 female nurses completed the study survey (50.2% and 49.8% of the nurses were married and unmarried, respectively). The largest age group (51.3%) ranged from 31 to 40 years, 33.8% of participants were between 20 and 30 years of age, and 14.8% were between 41 and 60 years of age. Most of the study participants were non-Saudi and accounted for 69.6% of the study population, with 71.9% of these nurses working in specialty areas such as the pediatric intensive care unit, the neonatal intensive care unit, and the emergency room. Regarding working shift hours, more than half of the participants (58.2%) were covering 12 h shifts and 41.8% worked 8 h shifts. Regarding the educational level, a considerable majority (82.1%) of the surveyed staff nurses had a bachelor’s nursing degree, whereas 17.9% had a diploma certification as their highest qualification. Concerning the years of clinical experience, 33.8% of the nurses had worked for 1–4 years, 32.3% for 5–9 years, and 33.8% for 10–15 years. See Table 1.

### 3.2. Rating PES Dimensions and Items

In this study, the mean average PES score for the four dimensions across the 12 items was high (*M* = 67.9, *SD* = 15.3). The mean score for the meaning subscale was the highest (*M* = 17.7, *SD* = 4.05), whereas the lowest mean score was obtained in the self-determination subscale (*M* = 16.2, *SD* = 3.97; Table 2).

In terms of scores for PES items, the statement “I am confident about my ability to do my job” had the highest mean score (*M* = 5.99, *SD* = 1.39), followed by “The work I do is meaningful to me” (*M* = 5.94, *SD* = 1.40) in the competence and meaning subscales, respectively. By contrast, the item “I have a great deal of control over what happens in my department” had the lowest scores (*M* = 5.25, *SD* = 1.39), followed by “I have considerable opportunity for independence and freedom in how I do my job” (*M* = 5.30, *SD* = 1.34) in the impact and self-determination subscales, respectively.

### 3.3. Differences in the PES and Personal Work Profile Scores

Regarding personal work profiles, independent sample *t*-tests were conducted. This test was statistically significant only between the PES and its subscales for the non-Saudi nationality variable (*M* = 70.10, *SD* = 15.25, *t* = 3.50, *p* = 0.001; Table 3). Additionally, the one-way ANOVA was conducted between PES and personal work profiles. Table 3 shows the significant difference between the PES scale/subscales with significant variables and the PES scale with other insignificant personal-work-profile variables.

## 4. Discussion

This study has found high PE levels and low levels for the dimension impact, similar to the results reported in Saudi Arabia [19], China [10], New Zealand [13], and Iran [16] but lower compared to findings in a US study [30]. The high PE score level may be due to most study participants having an educational level of a bachelor’s degree or above and working in a specialty area. It has been reported that PE scores are higher among those with a master’s degree [16]. Differences in management styles across hospitals may have influenced the perception of psychological empowerment [11]. Moreover, it has been suggested that nurses with access to support, recourses, opportunities, and information systems are more confident and independent [11]. Additionally, the work environment is a significant direct positive predictor of PE [12], although caution may be warranted. The most interesting finding of our study was that the PE dimensions meaning and competence yielded the highest scores. These findings are supported by results from previous studies [10,11,30]. This suggests that nurses working in pediatric units have the knowledge and skills to provide patient care as the majority of the participants worked in specialty areas that required a set of master skills and knowledge, although caution may be applied here.

The most prominent finding to emerge from this study is the significant difference between PE and nationality which is consistent with the results of a previous study [10]. The current study revealed that registered nurses with more working years and non-national nurses were more psychologically empowered. Moreover, the PE dimensions meaning and competence yielded the highest scores. Most of the recruited Saudi nurses are fresh graduates at the beginning of their careers who are less experienced when facing work difficulties, challenges, and stress. Previous studies assessing the working conditions of Saudi nurses suggest that unsatisfying workplaces, work-related stress, ethical distress, and difficult working conditions may compromise the quality of patient care and the work-life quality and burnout of nurses [31,32,33]. However, recent evidence indicates that PE mediates the relationship between the quality of work-life factors and burnout [17]. Additionally, another study indicates that dissatisfaction with salary, workload, and teamwork is also associated with mental health distress, depression, anxiety, and stress in expatriate nurses [34]. However, these findings must be cautiously interpreted.

The study results further support the idea of implementing particular PE programs directed at Saudi nurses. Therefore, conducting research with an interventional design of programs that empower nurses may be effective. Similarly, Özbaş and Tel [15] reported that PE scores of oncology nurses increased after an intervention program. Additionally, a recent study reported the effectiveness of burnout intervention programs in reducing burnout among mental health nurses in Saudi Arabia [35]. This suggests that intervention programs can improve nursing practice. Surprisingly, one of the more important findings from this study was that no significant difference between PE and personal work profiles was detected, except for nationality. This is supported by previous findings that also found no significant relationships among PE, age, and level of education [14].

The ambitious plan in Saudi Arabia recently announced by the Crown Prince to provide support for overcoming the national nursing shortage focused on increasing the nursing workforce and nursing schools. However, we also have to focus on improving workplace conditions for the national nursing workforce. Therefore, improving the working conditions in the Saudi Arabian context influences, among others, health outcomes.

### 4.1. Limitations

The study is limited by a lack of information regarding the experiences of nurse managers and assistant nurse managers that reflects their essential role in creating an empowering work environment for pediatric nursing staff. In addition, an issue that was not addressed in this study is whether the participants’ responses were unbiased or subject to socially favorable responses with selection bias since only female nurses were study participants. Therefore, measures to prevent socially desirable responses should be implemented and considered in further studies. Despite its limitations, a generalization of the present findings is that the study adds to our understanding of the current status of PE among pediatric nurses in the Saudi Arabian context. Despite its exploratory nature, this study offered insights into some psychometric properties. The instrument demonstrated psychometric properties in a new, culturally adapted context, and the study results showed that the four subscales were highly reliable. The item analysis indicated that the items contributed to the instrument significantly, although the inter-item correlation within the subscales was high in some cases. Thus, further explorative and validation studies should be considered.

### 4.2. Practice Implications

In addition to the abovementioned points, hospital administration plays an important role in strengthening psychological empowerment in nurses through the four dimensions (competence, meaning, self-determination, and impact). This may relieve the impending national nursing shortage and enable nurses to act as leaders in clinical practice. Furthermore, hospital administrators can adopt updated approaches, such as implementing specific empowerment intervention programs and communication sessions where nurses can identify and express their needs, such as personal, family, and organizational perspectives. This could facilitate the empowerment of nurses, thereby retaining them.

## 5. Conclusions

This study set out with the aim of assessing the PES among registered nurses working in pediatric units and examined the significant difference between the PES and participants’ personal work profiles in the Saudi Arabian context. The study results revealed significant group differences in the PES and personal work profile scores. Moreover, the results suggested that the PES construct, based on Spreitzer’s theoretical framework, is not equivalent across nationalities and needs to be investigated more thoroughly to understand the perception of PE among national nurses. The psychometric properties of the PES were suitable for the new, culturally different context. PE measure was utilized in this study, which adds to the body of literature assessing local nursing, particularly pediatric nursing. Lastly, considering these implications, policymakers and hospital administrators may enhance and empower nurses. Overall, the study findings suggest that nurses may benefit from using more empowering strategies to enhance the nursing work environment.

## Figures and Tables

**Table 1:** Personal Work Profile of the Study Population (*n* = 263).

Variable	*N*	%
Gender		
Female	263	100
Marital status		
Unmarried	131	49.8
Married	132	50.2
Nationality		
Saudi	80	30.4
Non-Saudi	183	69.6
Age in years		
20–30	89	33.8
31–40	135	51.3
41–60	39	14.8
Educational level		
Diploma	47	17.9
Bachelor and above	216	82.1
Current working unit		
Specialty area	189	71.9
General area	74	28.1
Current working shift		
8 H	110	41.8
12 H	153	58.2
Years of nursing experience		
1–4 years	89	33.8
5–9 years	85	32.3
10–15 years	89	33.8

Note: H, hours number.

**Table 2:** Average Psychological Empowerment Scores/mean with Cronbach’s Alpha Coefficients.

Subscale/Scale	Score Range	Min	Max	Scores/Mean	*SD*
Competence	7–21	3.00	21.0	17.5 (5.88)	4.04 (1.34)
Meaning	7–21	3.00	21.00	17.7 (5.90)	4.05 (1.35)
Self-determination	7–21	3.00	21.00	16.2 (5.40)	3.97 (1.32)
Impact	7–21	3.00	21.00	16.3 (5.43)	3.95 (1.31)
PES total	7–84	12.00	84.00	67.9 (5.65)	15.3 (1.33)

Note: PES, Psychological Empowerment Scale; *SD*, standard deviation.

**Table 3:** Significant difference between PES Scale/Subscale Scores and Personal Work Profile Scores.

Variable		Scale and Subscales	*M*	*SD*	Statistical Test	*p*
**Nationality**:	Non-Saudi	PES Scale	70.1	15.2	*t* = 3.50 ^a^	<0.001 *
	Saudi		63.0	14.6		
**Nationality**:	Non-Saudi	Competence subscale	18.1	3.93	*t* = 3.86 ^a^	<0.000 *
	Saudi		16.1	3.93		
**Nationality**:	Non-Saudi	Meaning subscale	18.2	4.00	*t* = 3.39 ^a^	<0.001 *
	Saudi		16.1	3.89		
**Nationality**:	Non-Saudi	Self-determination subscale	16.7	4.02	*t* = 2.86 ^a^	<0.003 *
	Saudi		15.2	3.66		
**Nationality**:	Non-Saudi	Impact subscale	16.9	3.93	*t* = 3.32 ^a^	<0.001 *
	Saudi		15.1	3.74		
**Marital status**:	Married	PES Scale	68.1	16.8	*t* = 0.21 ^a^	<0.155
	Single		67.7	13.8		
**Educational level**:	Bachelor	PES Scale	68.1	16.0	*t* = 0.42 ^a^	<0.136
	Diploma		67.0	12.8		
**Unit type**:	Specialty Area	PES Scale	68.1	16.0	*t* = 0. 27 ^a^	<0.160
	General Area		67.5	12.0		
**Working hours**:	Up to 12 H	PES Scale	68.3	15.7	*t* = 0.55 ^a^	<0.578
	Up to 8 H		67.3	14.9		
**Age in years**:	20–30	PES Scale	67.7	14.2	f = 0.93 ^b^	<0.395
	31–40		67.2	15.7		
	41–60		71.0	16.7		
**Experience years**:	1–4 years	PES Scale	66.0	14.1	f = 2.03 ^b^	<0.133
	5–9 years		67.3	14.8		
	10–15 years		70.5	16.8		

* *p* < 0.05. PES, Psychological Empowerment Scale. H, hours, *M*, Mean. *SD*, Standard Deviation. ^a^ Independent sample *t*-test. ^b^ One-way ANOVA.

## Data Availability

The datasets used and/or analyzed during the current study are available from the corresponding author upon reasonable request.
